# Assessing the Cultural Fit of a Digital Sleep Intervention for Refugees in Germany: Qualitative Study

**DOI:** 10.2196/65412

**Published:** 2025-04-03

**Authors:** Maja Blomenkamp, Andrea Kiesel, Harald Baumeister, Dirk Lehr, Josef Unterrainer, Lasse B Sander, Kerstin Spanhel

**Affiliations:** 1 Institute of Psychology University of Freiburg Freiburg Germany; 2 Cognition, Action, and Sustainability Unit Institute of Psychology University of Freiburg Freiburg Germany; 3 Department of Clinical Psychology and Psychotherapy Institute of Psychology and Education Ulm University Ulm Germany; 4 Health Psychology and Applied Biological Psychology Institute of Sustainability Psychology Leuphana University Lüneburg Lüneburg Germany; 5 Institute of Medical Psychology and Medical Sociology Faculty of Medicine University of Freiburg Freiburg Germany

**Keywords:** Ukraine, eHealth, sleep disturbances, low-threshold treatment, culturally sensitive treatment, refugee, digital sleep, Germany, digital intervention, interview, content analysis, qualitative study, mental burden, mental health care, electronic health, digital health

## Abstract

**Background:**

Digital interventions have been suggested to facilitate access to mental health care for refugees, who experience structural, linguistic, and cultural barriers to mental health care. Sleep-e, a digital sleep intervention originally developed for German teachers, has been culturally adapted for refugees in Germany mainly coming from African and Middle East countries. With the increasing number of refugees from Ukraine and the associated diversity of cultural backgrounds among refugees in Germany, it is essential to assess whether existing digital interventions are culturally appropriate for this target group as well.

**Objective:**

The study aimed to investigate the perceived cultural appropriateness of Sleep-e in both its original and culturally adapted versions among refugees in Germany, hereby exploring and possibly contrasting the needs of refugees coming from Ukraine and other countries of origin.

**Methods:**

Overall, 13 refugees (6 from Ukraine, 23-66 years old; and 7 from other countries, 26-41 years old) participated in the study. Each participant went through parts of the original or culturally adapted version of the digital sleep intervention, with 5 participants going through both versions. A total of 17 semistructured interviews (11 for the adapted, 6 for the nonadapted intervention version) and 9 think-aloud sessions (6 for the adapted, 3 for the nonadapted intervention version) were conducted to assess cultural appropriateness, suggestions for adaptations, and perceived relevance. Data were transcribed, categorized, and analyzed using structured qualitative content analysis.

**Results:**

The findings showed key differences in the perceived appropriateness and identification between the 2 refugee groups and the 2 intervention versions. Ukrainian participants expressed positive (n=70) and negative (n=56) feedback on the adapted intervention version, which revealed identity conflicts, as the adapted intervention version was targeted at a refugee population with whom they could not fully identify (18 negative feedback quotes concerning the refugee example characters). Whereas they identified with the European context in the original version, they found the problems described less relevant to their experiences. In contrast, participating refugees from other countries found the culturally adapted version more comprehensible and culturally appropriate (55 positive and 5 negative feedback quotes). No significant usability issues were reported, but several participants highlighted the need for an individualization of the intervention content.

**Conclusions:**

Neither the original nor culturally adapted version of the digital sleep intervention fully met the needs of all refugee groups, highlighting the complexity of culturally adapting digital interventions for this population. Particularly, the identity conflict of participating Ukrainian refugees regarding the refugee context suggests that adaptation should go beyond regional considerations and consider the dynamics of social identity. These findings emphasize the relevance of including co-design processes with different refugee populations to ensure broad identification and, herewith, cultural appropriateness of digital interventions.

**Trial Registration:**

German Clinical Trials Register DRKS00036484; https://drks.de/search/de/trial/DRKS00036484

## Introduction

The number of people forced to seek refuge in new countries is increasing worldwide [1]. These people, hereafter referred to as refugees, are exposed to multiple stressors before, during, and after leaving their home countries, and they therefore experience increased psychological vulnerability and prevalence of mental disorders [2,3], including sleep problems [4]. However, there are major gaps in mental health care for refugees in the receiving countries such as Germany [5,6], resulting from a lack of (culturally sensitive) services, language barriers, differences in help-seeking behavior and residence status, as well as stigmatization, among other barriers [7-9]. To overcome these barriers, culturally adapted digital interventions focusing on transdiagnostic or somatically perceived symptoms are being discussed and investigated as low threshold offers for refugees [10,11]. In the general population, digital interventions show good effectiveness for a wide range of mental health conditions [12,13]. Their usability appears to be essential in this context [14,15]. When offered to refugees, in addition to intervention usability, cultural adaptation is suggested to be essential for the acceptance and effectiveness of face-to-face and digital interventions, recognizing the specific burdens associated with the refugee context [16-18]. In this context, the question of the cultural specificity of the adaptations is becoming increasingly important [19]. The target group of refugees is characterized by a diversity of countries of origin that is constantly changing due to events in the global political situation, such as the start of the war in Ukraine in 2022. To increase refugees with diverse backgrounds using and profiting from digital interventions, these interventions need to be provided in a culturally sensitive (ie, with an openness to diverse backgrounds)—in contrast to a culturally specific (ie, separately for diverse backgrounds)—way [20]. Yet, so far, there are only a few digital interventions developed for refugees from diverse countries of origin [21-23].

Based on feedback from refugees in Germany, who at the time were mainly coming from African and Middle Eastern countries of origin, the digital sleep intervention Sleep-e was culturally adapted for this target group with the aim of making it accessible to people sharing the experience of flight. Thereby, (1) factors related to the flight (eg, separation from the family and the home country) and (2) factors related to the habits, values, and treatment concepts of their non-Western countries of origin were considered [24]. The culturally adapted version of the digital sleep intervention was accepted by refugees from African and Middle Eastern countries [22]. Based on the increasing number of refugees in Germany coming from Ukraine since the beginning of the war in Ukraine in 2022 [25], it seems essential to make such interventions available to this target group as well. Ukrainian refugees are shown to differ from refugees from other countries of origin, among others, in terms of sociodemographic aspects (age and gender), sociocultural aspects, differences in the flight route, legal rights, and welcoming culture upon arrival in Germany [26-29]. Building on the named 2 previous studies [22,24], this qualitative study aimed to investigate differences in the perceived cultural appropriateness and the cultural needs in regard to the digital sleep intervention among refugees from Ukraine and refugees from other, predominantly African and Middle Eastern countries of origin. A culturally adapted version and a nonadapted version of the intervention were used, and factors influencing the perceived cultural appropriateness were additionally investigated.

## Methods

### Recruitment and Participants

This study was conducted in 2 subsamples: refugees from Ukraine and refugees from countries other than Ukraine. Recruitment was carried out from February to April 2023 via various institutions and associations working in the refugee and migration context in Germany, mainly Freiburg and Karlsruhe. Many recruitment inquiries remained unanswered (eg, integration and language courses, migration offices, advice centers, and advertising via social media). Recruitment mainly succeeded via Refugium Freiburg (psychosocial advice for refugees), the German-Ukrainian Choir Karlsruhe, the University of Freiburg, the radio project “Our Voice” for refugees in Freiburg, and “Kontaktcafé Freiburg” (get-together café for refugees), as well as via personal contacts. Institutions were contacted consecutively face-to-face, via phone, or via email. They forwarded a flyer with information on the study to their clients face-to-face. People interested in study participation could contact the study team via email or phone and were then forwarded the detailed study information. Inclusion criteria were checked, which included the experience of flight, age of majority, and sufficient knowledge of German or English. No cutoff for insomnia severity was used as an inclusion criterion in order to also reach refugees with subclinical symptoms, who could be a key target group for the transdiagnostic and low-threshold digital sleep intervention. In addition, this allows information to be obtained on who might be interested in the intervention, regardless of existing clinical symptoms. Contacted participants who did not want to participate in the study mainly reported reasons such as language barriers or difficulties in finding the time for study participation. None of the participants dropped out after signing the informed consent.

There were 6 participants from Ukraine (23-66 years old), and 7 participants coming from Afghanistan, Gambia, Syria, Madagascar, Cameroon, Ecuador, and Eritrea (26-41 years old), referred to below as refugees from other countries. On average, the participants revealed subthreshold sleep problems. Detailed sociodemographic data are shown in Table 1.

**Table 1:** Sociodemographic data and information on the conduction of the qualitative study in Germany in 2023 are illustrated for each participating refugee from Ukraine (ukr) or other (oth) countries of origin.

Subgroup and ID			Home country (gender)	Education		Age (years)		Years in Germany		Psychotherapy (current or past)		Insomnia severity^a^		Tested modules		Study mode (study language)		Duration (min)		Codes, n	
**Ukraine**																					
	01_ukr	Ukraine (female)		High school	45		1		No		8		na^b^1: qtn^c^, int^d^ a^e^1: ThA^f^, int		In person (German)		79		154		
	02_ukr	Ukraine (female)		High school	45		1		No		15		na2: qtn, ThA, int a2: int		In person (German)		53		57		
	03_ukr	Ukraine (female)		High school	66		1		No		7		na3: qtn, ThA, int a3: int		In person (English)		57		105		
	04_ukr	Ukraine (female)		High school	26		1		Yes		15		a2: qtn, ThA, int		Video call (English)		42		65		
	05_ukr	Ukraine/Nigeria (male)		High school	23		1		No		15		a3: qtn, ThA, int		In person (English)		63		108		
	06_ukr	Ukraine (female)		High school	33		0.5		Yes		19		a4: qtn, int		Video call (English)		15		42		
	Total, mean (SD)	—^g^		—	39.7 (15.9)		0.9 (0.2)		—		13.2 (4.7)		—		—		—		—		
**Other countries**																					
	07_oth	Afghanistan (male)		High school	41		8		No		9		na1: qtn, int		Video call (German)		23		18		
	08_oth	Gambia (male)		No degree	27		3		Yes		18		na2: qtn, int a3: int		In person (English)		45		71		
	09_oth	Syria (male)		High school	26		7.5		Yes		8		na3: qtn, ThA, int a4: ThA, int		In person (German)		74		125		
	10_oth	Madagascar (male)		High school	30		10		Yes		18		a1: qtn, ThA, int		In person (French)		37		31		
	11_oth	Cameroon (female)		High school	33		1.5		No		6		a2: qtn, int		Video call (French)		35		13		
	12_oth	Ecuador (male)		High school	32		0.5		No		15		a3: qtn, ThA		In person (German)		33		18		
	13_oth	Eritrea (male)		No degree	31		8		Yes		12		a4: qtn, int		Video call (German)		11		23		
	Total, mean (SD)	—		—	31.4 (4.9)		5.5 (3.7)		—		12.3 (4.9)		—		—		—		—		

^a^Insomnia Severity Scores [30]: 0-7, not clinically significant; 8-14, subthreshold; 15-21, clinical (moderate); 22-28, clinical (severe).

^b^na: nonadapted version of the digital sleep intervention (modules 1-3).

^c^qtn: questionnaire.

^d^int: interview.

^e^a: adapted version of the digital sleep intervention (modules 1-4).

^f^ThA: think-aloud.

^g^Not applicable.

### Procedure

Data collection took place in one-to-one sessions (approximately 90 minutes of video-based or in-person, one-to-one meetings at the Institute of Medical Psychology and Medical Sociology, University of Freiburg, Germany) with MB, who was in the final year of her master’s degree in clinical psychology at the time of study conduction and had previous experience in qualitative research (refer to Table 1 for further information). MB had previous contact with one participant (01_ukr); the other participants were unknown to her. The participants were informed that the research was part of MB’s master thesis. Sessions were conducted in German, English, or French and were audio recorded. All participants completed a web-based questionnaire (sociodemographic data, Insomnia Severity Index with 7 Likert-scale items 0-4 [30], and Client Satisfaction Questionnaire adapted to internet-based interventions with 8 Likert-scale items 1-4 [31]). To assess the perceived cultural appropriateness of the respective interventions as part of the usability test, it was considered sufficient to work through individual modules of the adapted versus nonadapted intervention: many aspects relevant to the assessment are similar across the respective adapted versus nonadapted modules (eg, number of pictures, use of text versus video, and example characters with a German versus another cultural background). Following this assumption, the participants worked through one adapted or nonadapted module. They were then offered the opportunity to test a second (nonadapted or adapted) module. Three participants from Ukraine and 2 participants from other countries completed both an adapted and a nonadapted module. When assigning the modules, it was ensured that each module was seen at least once by participants from each subgroup. After each module completion, semistructured interviews were conducted in order to assess the participants’ experiences with the content and design of the tested module, its appropriateness for themselves and refugees in general, and their own usage behavior (refer to Multimedia Appendix 1 for the interview guideline; as the interview guideline was shown to be appropriate in a first pilot interview, the pilot was included in the final results). The think-aloud method [32] was additionally used with 8 participants (5 participants from Ukraine, and 3 from other countries), depending on the language skills and time resources of the respective participants. Using this method, the participants were asked to speak out loud all of the thoughts they had while they went through the modules; for example, they could comment on the experienced design, functionality, or content of the intervention. Throughout data collection, the investigator made observational notes on verbal or nonverbal characteristics during the test. Table 1 shows in detail which modules were processed by which participants and in which format (interview or think-aloud) the data were collected.

### Intervention

Two versions of the digital sleep intervention Sleep-e were used, which is a digital, brief cognitive behavioral intervention for sleep problems available in English and German. The intervention was provided on the eHealth platform eSano. The nonadapted version [24] is an unguided digital intervention consisting of 3 content-separated, consecutive modules based on the intervention GET.ON Recovery [33,34], which was developed and evaluated for German employees with sleep problems. Adaptations were conducted based on feedback from health care professionals working with refugees as well as refugees from Algeria, Eritrea, Iran, Iraq, and Syria [24], complemented by previously conducted cultural adaptations of digital interventions [35]. The resulting culturally adapted version consists of 4 content-separated, consecutive modules and was aimed at being suitable for refugees from diverse countries of origin in Germany, with the idea of a shared experience of pre-, peri-, and postmigration stressors. Its feasibility, acceptance, and preliminary effectiveness were investigated among refugees mainly coming from African and Middle East countries [22]. Table 2 provides information on the content of both versions of the intervention. Information on the adaptations suggested [24] and conducted [22] can be found in the respective articles.

**Table 2:** Overview of the modules of both an adapted and a nonadapted version of a digital Sleep-e intervention tested in the qualitative study with refugees in Germany.

Module	Content of the nonadapted version [24]	Content of the culturally adapted version [22]
Module 1	Introduction of example characters from Germany ("text-based"^a^ with photo) Psychoeducation and exercise on sleep hygiene rules ("text-based", quiz) Psychoeducation on sleep medication ("text-based") Optional: sleep diary ("offline document")	Introduction by a health expert and for example characters from Arabic-speaking countries ("video-based") Psychoeducation on sleep problems, link to "migration" ("video-based") Optional: relaxation exercise (audio-based) Optional: sleep diary ("short web-based document")
Module 2	Psychoeducation on the topic of rumination ("eg, explanatory video") “3” exercises on the topic of rumination (audio- and text-based)	Psychoeducation and "optional" exercise on sleep hygiene ("mainly video-based") "Optional": psychoeducation on sleep medication ("text- and video-based") "Optional": relaxation exercise ("audio-based") "Optional": sleep diary ("short web-based document")
Module 3	Reflection on the results, consolidation of what has been learned (text-based) Planning of future exercises ("text-based") Information on further offers of help ("text-based") Psychoeducation on problems associated with poor sleep ("text-based")	Psychoeducation on the topic of rumination, focus on migration topics (mainly video-based) “Optional”: 2 exercises on the topic of rumination (audio- and text-based) “Optional”: exercise on sleep hygiene rules “Optional”: relaxation exercise (audio-based) “Optional”: sleep diary
Module 4	—^b^	“Optional”: psychoeducation on problems associated with poor sleep (mainly video-based) Information on further offers of “refugee-specific” help “Optional”: exercise on sleep hygiene rules “Optional”: relaxation exercise (audio-based) “Optional”: reflection on results “Optional”: sleep diary

^a^Differences between the adapted and the nonadapted version of the digital sleep intervention are in double quotes*.*

^b^Not applicable.

### Data Analysis

Before analyzing data, the authors involved in the analysis (MB and KS) reflected on their own assumptions regarding the topic of the study. The following assumptions were identified: (1) refugees are often not well off and are grateful for any form of support and (2) Ukrainian refugees and refugees from other countries of origin differ in their needs. The participants were not involved in data transcription, analysis, or interpretation.

The collected data comprised 26 audio recordings (9 think-aloud sessions, including one participant who did 2 sessions; 17 interviews, including 5 participants who did 2 interviews). The recordings were automatically transcribed using Adobe Premiere (2021) and postprocessed by MB based on the transcription rules for computer-assisted analysis according to Kuckartz and Rädiker [36]. The data were analyzed using structured qualitative content analysis (primary analysis [36,37]) with a combination of content-related, evaluative, and contrasting categories [38]. MAXQDA 2022 [39] software was used to code a total of 830 text passages. The categories were created and continuously adapted by MB using an inductive and deductive hybrid approach [38], primarily based on the transcripts of the Ukrainian participants. This enabled the deductive inclusion and validation of theories and questions derived from the two previous studies, as well as the inductive inclusion of new aspects (eg, in regard to the war in Ukraine) introduced by the participants. After coding half of the transcripts (ie, 13), the new information could be integrated into the previously formed categories and no further changes were made to the structure of the category system, which indicates data saturation. In order to assess intercoder reliability and ensure the comprehensibility of the category definitions, 2 transcripts were additionally coded and discussed by KS (researcher at the time of study conduction). The different category formats were used to identify possible group differences (contrasting categories), evaluations (evaluative categories), and factors influencing the evaluations (content-related categories), as well as their interactions. As part of the secondary analysis, valence analyses [37] were used to compare the ratings of the intervention parts based on the evaluative categories (positive vs negative feedback regarding the adapted vs nonadapted intervention or regarding refugees from Ukraine vs from other countries of origin). In addition, the coding frequencies by categories, subgroups, and interventions were compared in frequency analyses, and overlaps between evaluative categories and contrasting or content-related categories were examined in group comparison. Such frequency analyses can provide insight into possible correlations but are of limited scalability due to the lack of objectivity of the number of codes. The qualitative results are shown along the developed category system, underpinned by quotes from the participants. The references of the quoted text passages comprise the number of participants (01-13), the subgroup (coming from Ukraine [denoted as “ukr”] vs from other countries of origin [denote at “oth”]), the modules processed (modules 1-4 of the adapted version of the intervention [denoted from “a1” to “a4”], modules 1-3 of the nonadapted version of the intervention [denoted from “na1” to “na3”]), the type of assessment (think-aloud [denoted as “ThA”], and interview [denoted as “int”]) and the position in the transcript (denotes as “pos.”). The quantitative data from the questionnaires was analyzed descriptively.

### Ethical Considerations

The study was registered in the Freiburg Clinical Trials Registry (registration FRKS004288). Ethics approval was granted by the ethics committee of the University of Freiburg, Germany (22-1452-S2). The study was conducted and documented in accordance with the guidelines for research with refugees [40] and the COREQ (Consolidated Criteria for Reporting Qualitative Research) [41] (refer to Multimedia Appendix 2 for the checklist). Interested participants received detailed digital, written information on the study procedure, aim, and their rights. Afterward, they provided their digital informed consent before the start of the study or had the option of withdrawing study participation without any consequences. The protection of the privacy and confidentiality of the participants was ensured by pseudonymizing all collected data with an ID. Only the pseudonymized data were used during the study, and the data were only accessible to selected project members. A reference list, which linked personal information with the ID, was deleted at the end of the data collection. This means that all data collected (transcripts or questionnaires) are de facto anonymized, and it is not possible to draw any conclusions from these data about the individual study participants. The de facto anonymized data of the participants are stored by the researchers on secure servers for 10 years after publication and then deleted. The participants had the chance to win one of five €20 (€1=US $1.10) gift vouchers as compensation for their efforts.

## Results

### Overview

The developed category system (refer to Multimedia Appendix 3) resulted in 6 main and 14 subcategories. In total, 4 main categories informed the first research question and contained information on the perceived cultural appropriateness and the cultural needs among the participating refugees from Ukraine versus those from other countries of origin regarding the digital sleep intervention: (1) concept: adapted version, (2) concept: nonadapted version, (3) comparison: adapted and nonadapted version, and (4) use of the intervention. The developed category system furthermore resulted in 2 main categories informing about factors that influenced the perceived cultural appropriateness and needs of the participating refugees regarding the digital sleep intervention: (1) identity and (2) health care.

### Perceived Cultural Appropriateness of the Digital Sleep Intervention

#### Overview

**Figure 1:**
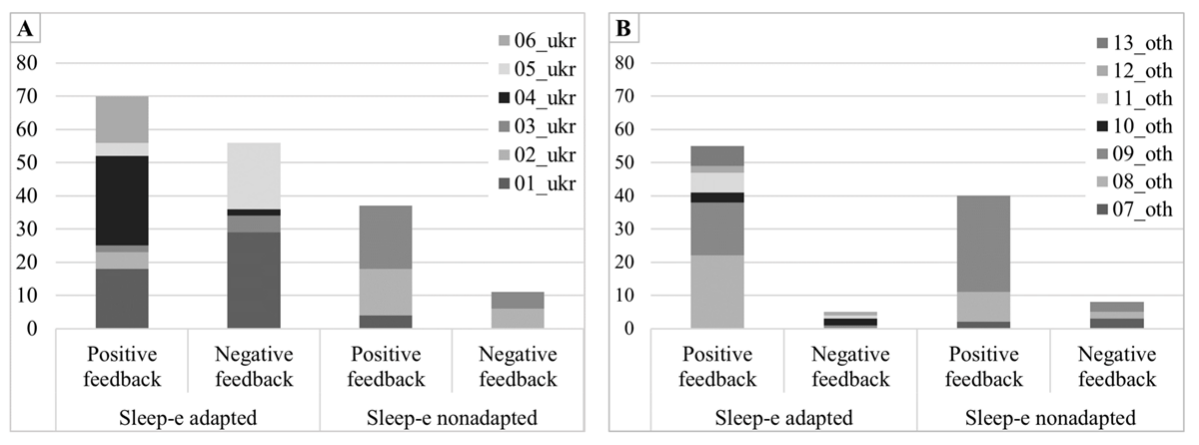
Frequency analyses of positive and negative feedback given by the refugees from (A) Ukraine and (B) other countries of origin participating in the qualitative study in Germany. The numbers of positive and negative feedback are illustrated separately for the adapted version and the nonadapted version of the digital intervention Sleep-e tested in the study. oth: other countries of origin; ukr: Ukraine.

Across both versions of the intervention and both subgroups, there was more positive (n=202) than negative feedback (n=80; see Figure 1).

You can take a lot from it also. And you can learn easily also.

08_oth_a3(na2)_int, pos. 211

To have the training..., it’s much easier. Yeah, and it could solve your problem.

06_ukr_a4_int, pos. 60

An overall positive attitude also was revealed in the relatively high ratings in the client satisfaction questionnaire adapted to internet-based interventions, with an overall mean score of 26.0 (SD 4.7) and similar mean scores for the different versions or subgroups.

#### Adapted Intervention Version

The adapted version of the intervention was rated positively in both groups, but the feedback from the Ukrainian participants revealed a rather heterogeneous picture, with 70 positive and 56 negative feedback quotes, contrasting with 55 positive and 5 negative feedback quotes among participants from other countries. With regard to the conception of the intervention, linguistic simplifications (eg, subtitles), the video formats and quizzes, as well as good structuring and feasibility were considered particularly helpful. Both groups criticized the quality of the videos as well as linguistic aspects such as the length of the texts and modules or language difficulties. As for the content of the intervention version, the experience reports from the example characters (refugees) in particular were described as motivating and interesting and the exercise content as helpful. Differences in the perceived appropriateness of the content between the 2 subgroups were found with regard to the identification with the example characters: whereas the participating refugees from other countries of origin were able to recognize and name similarities with the example characters, the participants from Ukraine did not identify with the example characters and described them as untrustworthy or boring (“I don't believe them that the exercises help.” [01_ukr_a1na1_int, pos. 102, translated]). In addition, the participants from Ukraine criticized the strong focus on the topic of flight, too negative content, and too little concrete advice. Suggestions for changes to the adapted version of the intervention included ideas for increasing identification, for example, through the opportunity to create a personalized profile with one’s own needs and issues (“Maybe bubbles and then it’s like: hit which bubble you feel speaks to you.” [05_ukr_a3_int, pos. 336]; “Create your own profile and then really see: voilà, that’s me.” [10_oth_a1_(ThA)int, pos. 147, translated]). According to the participants (especially those from Ukraine), this could also be achieved through a greater variety or abstractness (eg, by using graphics instead of real videos) of the example characters.

#### Nonadapted Intervention Version

The nonadapted version of the intervention was also rated positively in both groups (37 positive and 11 negative feedback quotes from the Ukrainian participants; 40 positive and 8 negative feedback quotes from participants from other countries). With regard to the conception of the intervention, its simple design and the clear, motivating, and comprehensible structure were mentioned. Both groups criticized the difficult language and an oversupply of text-based information. As for the content of the intervention version, the experience reports of the example characters (Germans) and the exercises and questions were described as helpful, as was the combination of theoretical and practical aspects (“So the first [nonadapted] version was technical for me. Going to see a therapist and answering questions I could fully understand and that was professional I would say.” [01_ukr_a1na1_int, pos. 6, translated]). However, the content of this version of the intervention was partly considered too scientific or insufficient for more serious problems by the participants from Ukraine (“I don’t see here any problems, serious problems. Just, time to bed, stop having alcohol, stop working after 9. But people who are under stress, need the more serious program*.*” [03_ukr_na3_int, pos. 123]), whereas the participants from other countries of origin mainly mentioned identification difficulties (“Except for alcohol and so on, because I'm a Muslim and I don’t drink alcohol. Yes, and the norm in the Arab-Islamic world is completely different. It’s practically a sin, it’s an absurdity, alcohol is for losers or sinners, or I don’t know, and not for normal people.” [09_oth_na3a4_int, pos. 57, translated]). Some statements of the Ukrainian participants did not include themselves when pointing out possible limitations of the cultural appropriateness for refugees (“But not all people can read so much text at once and the language is also a bit too scientific, like from a psychological book [...] For me it was okay.” [02_ukr_na2_ThA_int_a3_int, pos. 39, translated]). Suggestions for changes to the nonadapted version of the intervention included a more solution-oriented content and the presentation of more appropriate problems or situations (“I would yes, more different [example characters], for example in terms of age.” [07_oth_na1_int, pos. 111, translated]).

### Factors Influencing the Perceived Cultural Appropriateness

As a main factor influencing the perceived cultural appropriateness of the intervention, identification was extracted. In this context, the participants emphasized the need to enhance identification with the intervention by including possible differences in the refugee contexts as well as cultural and linguistic differences in the intervention content.

#### Identification With the Refugee Context

Factors related to the refugee background and the identification with it were shown to influence the perceived appropriateness of the interventions among both groups of participating refugees. Some of these factors were related to their own flight experiences (“Especially when they came from Ukrainian zones after the bombing, after devastation, ruin, these bad things. They ask to get psychological treatment or to see a psychiatrist because they realize they need it.” [03_ukr_na3_int, pos. 72]; “That is another traumatic experience. Knowing that crossing the sea, some people die, some people survive.” [08_oth_a3(na2)_int, pos. 153]).

Yet, a separation of the participating Ukrainian refugees from being a refugee became apparent, revealing an underlying identity conflict (identity of being a refugee): “They don't identify as refugees, so in a way that [experience reports from the adapted version of the intervention] would even be a disconnect for them.” [05_ukr_a3_int, pos. 246]; “Just I lived in prosperity [...] I have everything. And I fled from Ukraine the second day of war and didn’t see all those bombings, devastations, and so on and so forth.” [03_ukr_a3_(ThA)int, pos. 11]. Along with this, the Ukrainian participants emphasized that they did not want to be confronted with the topic of flight (“If I were a refugee, I wouldn't want to focus on it...You have to forget that you are a refugee.” [01_ukr_a1na1_int, pos. 243, translated]).

Along with the identification as a refugee, the question of how far the identity of being a refugee is compatible with the Ukrainian identity was revealed, and whether refugees from Ukraine can identify with refugees from other countries of origin (same vs different identity). Thereby, statements about the same versus different identities were repeatedly raised (Textbox 1).

**Statements by the participating refugees in the qualitative study conducted in Germany about the same versus different identities of refugees from Ukraine (ukr) and refugees from other countries of origin (oth).**

- “You have a world with Ukrainians inside who do not just view fleeing the same, you know, it's the same war, but at the same time, it is not the same (laughs).” [05_ukr_a3_int, pos. 252]
- “There are universal factors of flight and war.” [09_oth_na3a4_int, pos. 306, translated] versus “It is never the same experience.” [05_ukr_a3_int, pos. 300]
- “The people that came from the other places also, they face even more difficulties than the people that came from Ukraine.” [08_oth_na2a3_ThA_int, pos. 353] versus “First-degree refugees, second-degree refugees...as if they were now downgraded, that is, with the refugees who are now less privileged.” [09_oth_na3a4_int, pos. 342, translated]
- “They have a different pronunciation, different skin color, different way of speaking and different challenges than we do.” [09_oth_na3a4_I, pos. 336, translated] versus “They are also refugees and they have had all these problems.” [01_ukr_a1_T, pos. 432, translated]

The dilemma of same versus different identity also became evident in various statements regarding a possible identification of the participating refugees from Ukraine with the example characters in the adapted version of the intervention: “When I was listening to these people, I did not, I do not know their situation.” [03_ukr_a3_(ThA)int, pos. 74] versus “I could relate. I understand his problem and I think that I have the same problem.” [04_ukr_a2_ThA, pos. 127] versus “Oh, [for refugees] from Ukraine. No.” [05_ukr_a3_int, pos. 240]. Various conflicting issues were found in their statements about the example characters (Table 3).

**Table 3:** Statements of the participating refugees from Ukraine can illustrate an ambivalent view on the example characters of the adapted version of the digital sleep intervention tested in the qualitative study in Germany.

Affirming perspective		Contrasting perspective
“To be honest, it is better for refugees.” [01_ukr_a1na1_int, pos. 6, translated]	↔	“Not for me. [laughs] For me, I don’t know, I understand that it’s a completely different culture.” [01_ukr_a1_ThA, pos. 432, translated]
“Of course, I don't have time to have this diary, because it's for people who are under stress and under problems...who have serious problems and they want to recover. They will do that.” [03_ukr_a3_(ThA)int, pos. 116]	↔	“Hm no, for me, stress is the problem number one...Too much stress, for sure. It can have a negative impact on the sleeping quality. Yeah, you can’t sleep because you think how to solve this problem.” [03_ukr_na3_int, pos. 18 // 03_ukr_na3_ThA, pos. 189]
“You know, it [experience reports from refugees]'s something you can relate to, you know.” [05_ukr_a3_int, pos. 234]	↔	“Even though I did not flee to Europe, like through the Mediterranean or the Sahara, fleeing the war in Ukraine, I don’t really think it speaks to me.” [05_ukr_a3_int, pos. 198]
“And besides: Being a refugee is difficult, it is always difficult.” [01_ukr_a1_ThA, pos. 170, translated]	↔	“No, for me, no, for me, everything is easy to talk about. I don’t have such or deep problems.” [01_ukr_a1na1_int, pos. 165, translated]

Related to this, frequency analyses of feedback from the participating refugees from Ukraine revealed 9 positive feedback quotes and 18 negative feedback quotes on the example characters in the adapted intervention version. In contrast, the participating refugees from other countries of origin seemed to identify strongly with the example characters in the adapted version of the intervention and did not mention any negative criticism of them, but gave 16 positive feedback quotes. Feedback on the example characters in the nonadapted intervention version was more homogeneous, with both 4 positive and negative feedback quotes given by the participating refugees from Ukraine and 3 positive and 1 negative feedback quotes given by the participating refugees from other countries of origin.

#### Identification With the Country of Origin

Other factors related to the flight background that influenced the perceived appropriateness of the intervention versions were associated with the identification with the respective country of origin versus the identification with Germany. This goes back to the fact of coming from non-German countries of origin, which was linked to revealed cultural or linguistic differences.

Because I'm Muslim and I don't drink alcohol.

09_oth_na3a4_int, pos. 57, translated

Nope, so the psychologists work according to models and views and worldviews that don't match mine.

09_oth_na3a4_int, pos. 243, translated

Also, a better linguistic understanding (native language, linguistic facilitation such as subtitles or audios, etc) and the consideration of different reading comprehension levels seemed to increase the perceived cultural appropriateness: “And not every refugee also can read. But having access to audible information can be helpful.” [08_oth_a3(na2)_int, pos. 137]. As such, the German language was perceived as stressful by one participant: “If I speak German, I'm tense. Yes, because it has to do with work, with problems, with documents and so on.” [01_ukr_a1_ThA, pos. 359, translated]. Whereas the participants from other countries of origin named both cultural and linguistic differences and identification issues, the Ukrainian participants solely named linguistic differences.

#### Health care

Besides the refugee identity, the factor “health care” was identified to be linked to the perceived cultural appropriateness of the digital sleep intervention. Differences in the needs and the use of mental healthcare became evident, revealing the importance of familiarity on the one hand and professionalism on the other hand.

I would opt for a European doctor.

01_ukr_a1na1_int, pos. 237, translated

That [nonadapted version] was professional I would say.

01_ukr_a1na1_int, pos. 6, translated

If you have problems, you'd rather go to mother, yes, [...] something like relatives, so your nation.

01_ukr_a1_ThA, pos. 491, translated

Although psychotherapy was partly described as “trendy” [02_ukr_na2_int, pos. 108, translated], stigmatization of mental health care became apparent: “Where I come from, it’s very taboo and really inaccessible.” [10_oth_a1_(ThA)int, pos. 129, translated]; this was also on a subconscious level (eg, observation from participant 11 [oth] when filling out the sociodemographic questionnaire: “Psychotherapy? Ah no no no!” [translated]).

## Discussion

### Principal Findings

#### Overview

**Figure 2:**
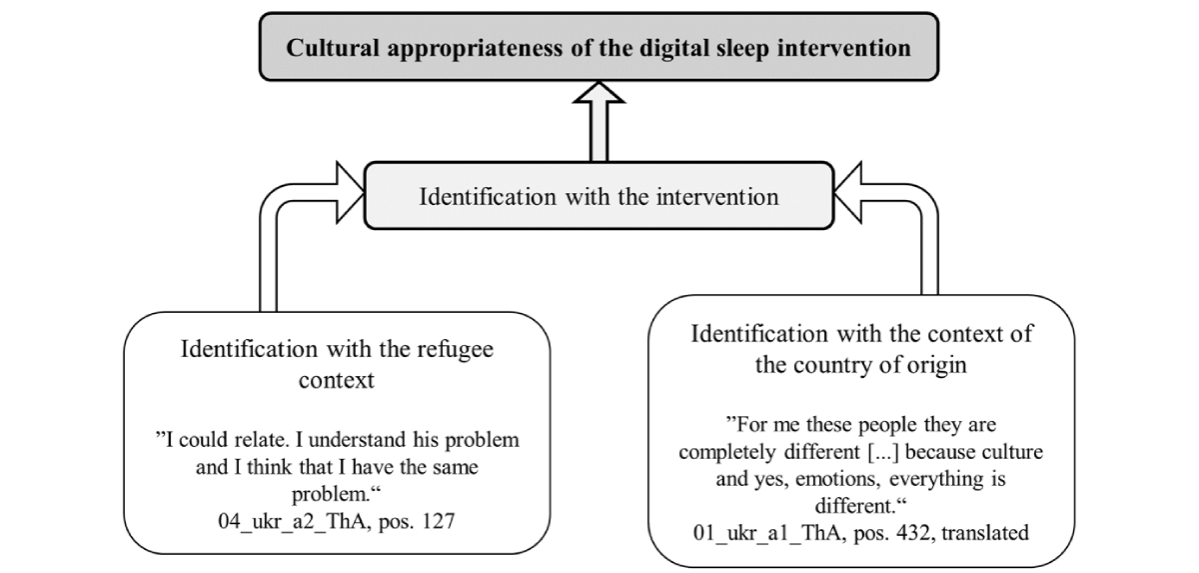
The role of identification in the cultural appropriateness of the digital intervention Sleep-e tested in the qualitative study in Germany, as perceived by the participating refugees from Ukraine and other countries of origin. a: adapted version of the intervention; pos: position in the transcript; ThA: think-aloud; ukr: Ukraine.

This study for the first time compared the cultural appropriateness perceived by refugees from Ukraine and other countries of origin regarding a digital intervention, specifically the original (developed for Germans) and the culturally adapted (adapted for refugees coming from African and Middle Eastern countries) versions of a digital sleep intervention. Overall, the participating refugees showed a positive attitude toward the intervention, yet the perceived cultural appropriateness regarding the 2 versions seemed to differ between the participants from Ukraine versus those from other countries of origin. The identification with the intervention content regarding the refugee context as well as the context of their countries of origin seemed to play a crucial role (refer to Figure 2). Identification with the refugee context refers to the extent to which participants see their own flight as a part of themselves and could relate to these issues in the intervention. Identification with the country of origin refers to the extent to which the participants identify with the behaviors, values, and norms of their country of origin and could relate to the behaviors, values, and norms illustrated in the intervention. Thereby, identity conflicts among the Ukrainian participants regarding the refugee context were identified as factors reducing the perceived cultural appropriateness of both intervention versions.

#### Differences in the Perceived Cultural Appropriateness of the Digital Sleep Intervention

Both versions of the digital sleep intervention received more positive than negative feedback, and feedback on less appropriate aspects mainly concerned design and language aspects (eg, including too much text and hard-to-understand language), which is rather general feedback on digital interventions than a refugee-specific point of view [42-44]. Regarding the participating refugees from countries other than Ukraine, the findings mainly corresponded to previous findings: whereas a lack of identification with the nonadapted version was shown [24], the adapted version seemed to be perceived as appropriate in terms of its content [22], and no negative feedback on the intervention content was given. Regarding the participating refugees from Ukraine, negative feedback on the intervention content was mainly given concerning the adapted version of the intervention and comprised a perceived overfocus on the topic of flight, in particular regarding the example characters. Concerning the nonadapted version, less negative feedback indicated a perceived inappropriateness of the illustrated life situations and problems of the example characters, which related in particular to a high workload. Thus, both versions of the intervention appeared to be only partially appropriate for the participants from Ukraine.

#### Identification Influences the Perceived Cultural Appropriateness of the Digital Sleep Intervention

The lower perceived cultural appropriateness among the Ukrainian participants regarding the adapted version of the intervention was suggested to be linked to their lower identification with the intervention: Differences between the participants from Ukraine versus those from other countries of origin became apparent with regard to the identification with the refugee context and aspects related to the identification with the own country of origin. This resulted in differences in the identification with, for example, the example characters in the interventions. The revealed differences correspond to differences between refugees from Ukraine and refugees from other countries of origin suggested in the literature: Although Ukrainian refugees and refugees from other countries of origin share the experience of having had to flee, they differ in various other aspects, such as the welcome culture in Germany as well as sociodemographics, which was also revealed in this study [27,45].

Along with this, higher privileges of Ukrainian refugees and differences in refugee experience or arrival in Germany were discussed among the participants. Not only are there differences between refugees from Ukraine and from other countries, but there are also major differences in the experiences of war and flight among refugees from Ukraine, particularly at the time the study was conducted, which was only one year after the beginning of the war [27]: Some of the participating refugees from Ukraine had left the country right at the beginning of the war without having experienced it themselves, and also without having experienced the loss of family members or friends. Furthermore, the Ukrainian participants might still have hoped to return to their home country soon [27]. Different durations of their stay in Germany therefore seemed to be linked to different flight experiences and, herewith, with a different identification with the refugee context and associated problems described in the adapted intervention version. Depending on this, the adapted version of the intervention could therefore show a certain degree of perceived appropriateness for some of the participating refugees from Ukraine. A dilemma became apparent: Whereas the Ukrainian participants seemed to identify with the European culture of the example characters in the nonadapted version, they did not identify with their concerns, especially regarding a high workload; with regard to the example characters in the adapted version, they could possibly identify in parts with their refugee background, but not with their non-European cultural background. This was accompanied by identity conflicts regarding the refugee context in the statements of the Ukrainian participants, which were not found among the participants from other countries of origin. For example, one Ukrainian participant described the adapted version of the intervention as appropriate for refugees, but not for herself. The importance of identity issues and conflicts in the refugee context has been highlighted previously [46,47].

The found differences in the identity conflicts and the identification with the versions of the intervention emphasize the relevance of specifying a target group and their specific needs in the context of cultural adaptations [48,49]. The lack of including the living situation and the needs of Ukrainian refugees during the cultural adaptation of the present intervention [24] may have led to the identification difficulties and, herewith, the reduced perceived cultural appropriateness. In order to enhance the appropriateness of the intervention for refugees from Ukraine, the intervention could be further adapted, for example by providing the possibility for personalization of the content or of the example characters in order to improve the identification. This has already been applied, for example, as part of the Step-by-Step program in Lebanon [50,51].

### Limitations

Several study limitations are to be named. First, the generalizability and robustness of the results are limited due to the qualitative and explorative character of the study. Furthermore, we could only include study participants with sufficient knowledge of German or English. Due to the open recruitment, this resulted in a rather educated and young sample, which possibly influenced the perceived cultural appropriateness of interventions [52]. Second, the majority of participants were recruited via private contacts. This may have biased the results in terms of social desirability, despite enquiring about perceived negative aspects of the interventions. Third, the duration and mode of conduct (video call vs in person) of the interviews with the study participants were not parallelized for the two subgroups. This might have led to an overrepresentation of the statements made by the Ukrainians, combined with the fact that the category system was initially developed based on the transcripts of Ukrainian participants. Fourth, the frequency analyses are not directly interpretable, as they were not related to the frequency of implementation of the respective intervention.

### Conclusions

With the rising number of refugees worldwide, the need for low-threshold and culturally appropriate mental health services is increasing. In this qualitative study, the relevance of the identification of the participating refugees with the content of the digital intervention Sleep-e for their perceived cultural appropriateness was emphasized. There were substantial differences between the participating refugees from Ukraine and those from other countries of origin: The participants from Ukraine appeared to be in an identity conflict regarding their identification with the refugee context, which seemed to reduce the perceived appropriateness both of the version of the intervention adapted for refugees from African and Middle East countries and of the nonadapted version for Germans. Refugees from other countries of origin, on the other hand, could identify well with the adapted version of the intervention and perceived it as appropriate. The perceived cultural appropriateness for refugees from Ukraine should be improved by diversifying the problems addressed to consider current developments related to the war in Ukraine and other changes in world affairs. As such, the cultural sensitivity of treatments should be considered as a dynamic condition related to time and context [53].

## Appendix

Multimedia Appendix 1 Interview guideline.

Multimedia Appendix 2 COREQ (Consolidated Criteria for Reporting Qualitative Research) checklist.

Multimedia Appendix 3 Category system.

## Abbreviations

COREQ: Consolidated Criteria for Reporting Qualitative Research

## Data Availability

The datasets generated or analyzed during this study are available from the corresponding author on reasonable request.
