# Supplementary material for: Assessing the Cultural Fit of a Digital Sleep Intervention for Refugees in Germany: Qualitative Study
Source: JMIR Form Res. 2025 Apr 3;9:e65412. doi: 10.2196/65412 (PMC12006774; doi:10.2196/65412)
Supplement: Multimedia Appendix 1 [file formative_v9i1e65412_app1.pdf]

Multimedia Appendix 1: Interview guideline

Narrative-generating question	Content/ memos	Potential further questions
**How would you evaluate the content of the training?**	Content	• Which of the components would you (rather not) work on?<br>• Which contents were missing in the training?<br>• What do you think the aim of the training should be?<br>• How did you feel about the examples given by the people in the training?
**How did you feel about the realisation of the training?**	Specifics in realisation/ design/ language	• What did you find successful about the realisation of the training?<br>• What would you do differently?<br>• How did you experience the language used in the training?<br>• Did you find the wording of the content understandable and appropriate?<br>• How did you feel about the design of the training?<br>• Were there any special features in the technical implementation of the training?<br>• How did you find the structure of the training?
**Were there parts of the training that you found inappropriate?**	Inappropriate content/ images	• Were there any images that you found inappropriate?<br>• Did you feel uncomfortable with certain parts of the training?
**Can you discuss whether such a training would be an option for you to seek help?**	Factors that facilitate uptake<br>Factors that hinder uptake	• Why? Why not?<br>• What would motivate you to do the training?<br>• What would make it easier for you to work through the modules?<br>• In what way or from whom would you like to hear about the training?<br>• What would hinder you from using such a training?<br>• What would cause you to stop using such training?<br>• What would you find very difficult to work through?

Narrative-generating question	Content/ memos	Potential further questions
**Would you feel comfortable in doing the training?**	Stigmatisation	• Why? Why not?<br>• If you were to use such training, would you be concerned that someone would approach you about it?
**Finally, what do you think about this kind of support (online sleep training)?**	Attitude	• What do you find good about this support option?<br>• What do you find bad about this support opportunity? (What concerns would you have about using the training?)<br>• Would you recommend the use of the training to others? Why? Why not?
**What do you think, is the training suitable for all people who have experienced fleeing from their home country?**	Flight	• Is it a good idea to do sleep training explicitly for refugees only?<br>• What special needs might people with refugee experience have for a sleep training? What factors of flight could influence this? Do you see differences here, e.g. between refugees from Ukraine and refugees from other countries?
