# Supplementary material for: Assessing the Cultural Fit of a Digital Sleep Intervention for Refugees in Germany: Qualitative Study
Source: JMIR Form Res. 2025 Apr 3;9:e65412. doi: 10.2196/65412 (PMC12006774; doi:10.2196/65412)
Supplement: Multimedia Appendix 3 [file formative_v9i1e65412_app3.pdf]

Multimedia Appendix 3: Category system

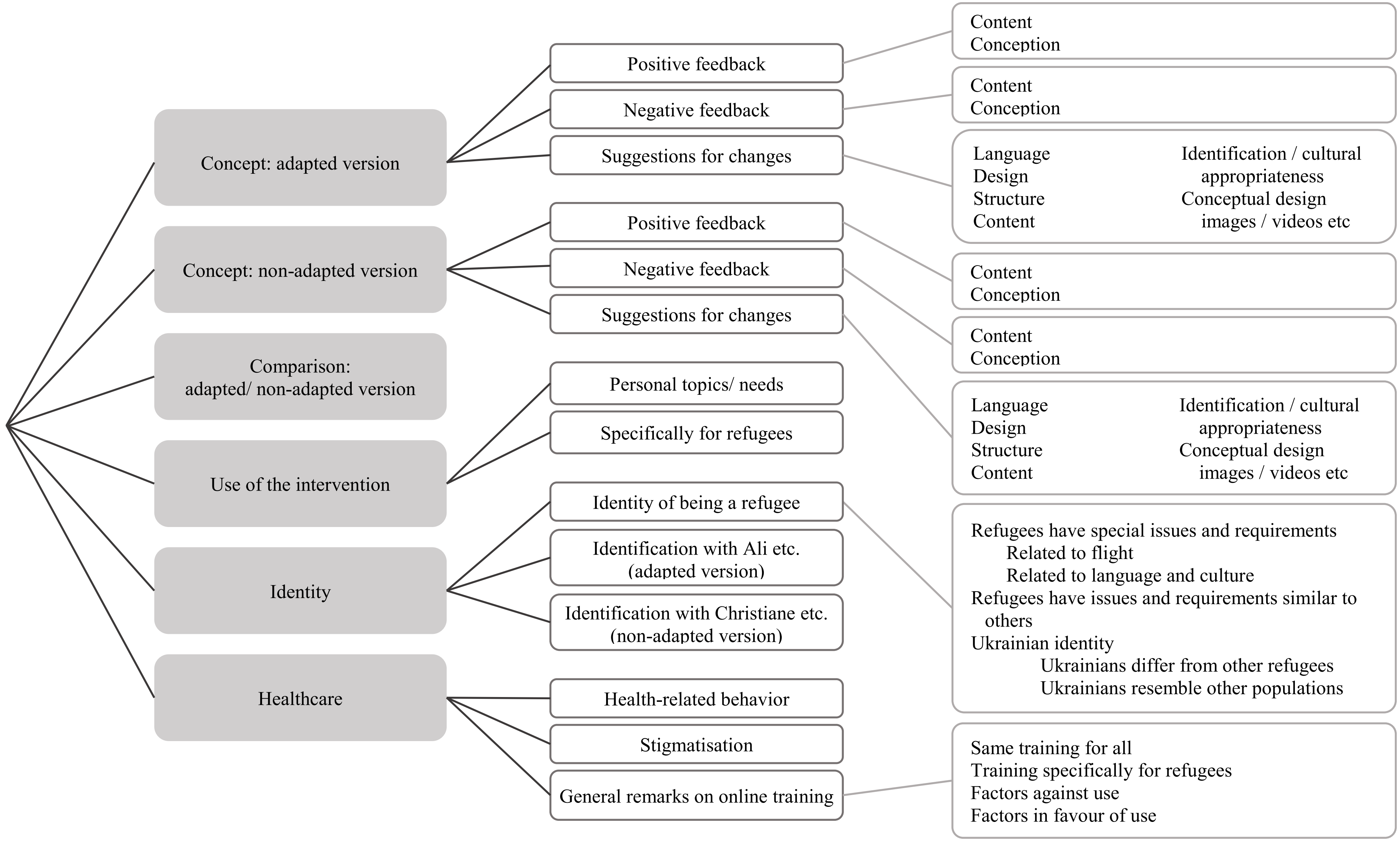

Category system derived from interviews with 13 participating refugees (26 transcripts) on an adapted version and a nonadapted version of a digital sleep intervention, processed through qualitative content analysis.
